# Stability enhancement of perovskite solar cells using multifunctional inorganic materials with UV protective, self cleaning, and high wear resistance properties

**DOI:** 10.1038/s41598-024-57133-8

**Published:** 2024-03-18

**Authors:** Seyyedeh Sedigheh Azad, Reza Keshavarzi, Valiollah Mirkhani, Majid Moghadam, Shahram Tangestaninejad, Iraj Mohammadpoor-Baltork

**Affiliations:** Department of Chemistry, University of Isfahan, Isfahan, 81746-73441 Iran

**Keywords:** Perovskite solar cells, AZO and ZnO, UV protective, Self-cleaning, High wear-resistance, Energy, Artificial photosynthesis

## Abstract

Organometal halide perovskite solar cells have reached a high power conversion efficiency of up to 25.8% but suffered from poor long-term stability against environmental factors such as ultraviolet irradiation and humidity of the environment. Herein, two different multifunctional transparent coatings containing AZO and ZnO porous UV light absorbers were employed on the front of the PSCs. This strategy is designed to improve the long-term stability of PSCs against UV irradiation. Moreover, the provided coatings exhibit two additional roles, including self-cleaning and high wear resistance. In this regard, AZO coating showed higher wear resistance compared to the ZnO coating. The photocatalytic self-cleaning properties of these prepared coatings make them stable against environmental pollutants. Furthermore, appropriate mechanical properties such as high hardness and low coefficient of friction that leads to high resistance against wear are other features of these coatings. The devices with AZO/Glass/FTO/meso-TiO_2_/Perovskite/spiro/Au and ZnO/Glass/FTO/meso-TiO_2_/Perovskite/spiro/Au configurations maintained 40% and 30% of their initial performance for 100 h during 11 days (9 h per day) against the UV light with the high intensity of 50 mW cm^-2^ which is due to higher absorption of AZO compared with ZnO in the ultraviolet region. Since AZO has a higher light transmission in the visible region in comparison to ZnO, perovskite cells with AZO protective layers have higher efficiency than perovskite cells with ZnO layers. It is worth noting that the mentioned features make these coatings usable for cover glass in all types of solar cells.

## Introduction

Perovskite solar cells (PSCs) have achieved a lot of attention in the past few years due to significant growth in their power conversion efficiencies (PCE) (from 3.8% in 2009 to 25.8% in 2021^1–5^. However, to commercialize PSCs, in addition to PCE, their stability must also be increased. Currently, the PSCs' stability is not meeting this target^6,7^. Unfortunately, the stability of PSC is limited by several environmental factors, mainly including ultraviolet light, high temperature, and humidity. Among these, the degradation induced by ultraviolet light remains challenging, and the environmental-deteriorating agents have been studied more^8–12^. Ultraviolet (UV) radiation is the most dangerous part of the sun's spectrum that causes PSC instability through perovskite degradation and increases the non-radiative recombination rate in PSCs-based mesoporous Titanium dioxide )TiO_2_)^7,13,14^. Titanium dioxide (TiO_2_) is the most effective electron transport layer (ETL) in PSCs. In recent years, the conducted research on PSC instability under UV light has shown that UV illumination decreases the performance and stability of PSCs in two ways^13,15,16^. First, upon shining ultraviolet light, because of the photocatalytic effect at the titanium dioxide (TiO_2_), methyl ammonium lead iodide (CH_3_NH_3_PbI_3_) perovskite photoactive layers rapidly decompose to PbI_2_ and CH_3_NH_3_I in an irreversible process. When TiO_2_ is exposed to UV light, because of the photocatalytic effect, it can extract electrons from the I^−^ ions in the perovskite and decompose it. Second, upon UV light exposure, photo-generated holes in the mesoporous TiO_2_ react with oxygen adsorbed at surface oxygen vacancies, which causes an increase in recombination and a decrease in cell performance^17–19^. To overcome these problems, various strategies have been porposed: (i) replacing mesoporous TiO_2_ as ETL with another ETL material like polyacrylic acid–stabilized tin(IV) oxide quantum dots, PbZrTiO_3_, ZnO-ZnS^20–22^ and; (ii) strengthening the perovskite/TiO_2_ interface like SrO, SnO_2_, andEu (TTA)_2_ (Phen) MAA^23–25^ and (iii) using luminescent down-shifting (LDS) to absorb short-wavelength light and re-emit them at a longer wavelength or utilizing UV absorbing layers on the front side of the devices to improve UV stability^26–29^.

Among the three mentioned approaches, the first and the second ones may cause to decrease in the performance of perovskite solar cells because of some issues, including the insufficient effect of TiO_2_ substitutions because TiO_2_ possesses a proper band gap and has no toxicity compared to others^21,24^. The third approach doesn't have the mentioned disadvantages.

UV-absorbing materials must have several features, including high transparency, high absorption in the whole UV light range, high photostability, and affordability, must be available. UV absorbers can be included inorganic and organic materials. So far, the materials used as the UV adsorbent layer before the TiO_2_ layer in PSCs often contained organic polymers^26^ and Eu compounds^28^ that had poor mechanical properties and low thermal stability, with no photocatalytic activity. However, Organic UV absorber application is severely limited due to the low stability and photodegradation upon UV irradiation^30,31^. Among the inorganic materials, metal oxide semiconductors improve the protective coating due to their highly efficient UV absorption, good transparency, and heat-resistance properties. Among nanoscale inorganic materials, zinc oxide (ZnO) with a wide band gap (Eg = 3.37 eV, corresponding to 376 nm) has unique electro-optical properties, effective absorptivity in the UV range, high light transmittance in the visible range, and excellent thermal-insulating efficiency. One of the other inorganic materials is aluminium-doped-zinc oxide (AZO), which is obtained by replacing Zn^2+^ atoms with Al^3+^. AZO semiconductors show UV and thermal stability in addition to, having enhanced electrical and optical properties compared to pure ZnO. These properties, along with high transparency, excellent photocatalytic activity, and mechanical properties such as scratch resistance, make ZnO and AZO good candidates as UV filters for perovskite solar cells^32,33^.

Notably, the UV absorption layers deposited on the front side of the perovskite solar cells can be a cover glass for all kinds of solar cells if they have appropriate mechanical and optical properties^34–36^.The first surface of the photovoltaic devices where interact with the incident photons of sunlight is cover glass, constituting a significant proportion of the device's cost^36^. Improvements in the performance of the cover glass in terms of mechanical and optical properties cause increasing module device lifetimes and PV module efficiencies. Mechanical resistance containing elastic modules, wear resistance, coating hardness^34–38^, and optical properties, including UV protection and high transmittance of light in the visible range, can be achievable by adding dopants to produce cover glasses^34,36,39^ Regarding the optical properties, Al doping to ZnO can reduce the structural defects and modify the crystal structure of ZnO, which leads to a decrease in the refractive index of the material. As a result, the AZO layer has a lower refractive index than pure ZnO, which means that it can let more light pass through the material^40–42^. On the other hand, one of the goals of this work was to prepare coatings with high wear resistance, which was provided by aluminum doping in the ZnO network. Since aluminum has higher hardness and tensile strength than zinc, adding aluminum to ZnO can increase the hardness and tensile strength of the AZO thin film and make the AZO thin film more resistant to deformation and wear than ZnO^43,44^. To determine the mechanical properties of thin films, nanoscratch, and nanoindentation are employed^34,38^. The most usual method used for the mechanical characterization of porous thin films is nanoindentation, which is used to determine the mechanical properties of thin films, such as hardness, stiffness, and module elasticity.

Further, the nanoscratch test can be used to determine the coefficient of friction (COF) and susceptibility to wear of thin films. To receive the maximum solar power conversion efficiency, keeping clean solar panel surfaces, such as reducing the soiling of the interface, is essential. Utilizing self-cleaning surfaces through the photocatalytic reaction instead of traditional methods for the degradation of various pollutants is a suitable approach in this context^36,45^.

In this work, two kinds of multifunctional coatings comprising ZnO and AZO inorganic UV absorber materials are experimentally deposited and optimized on the glass side of the FTO substrate (FTO 15Ω sq^–1^) in a PSC device. These multifunctional coatings exhibit several properties simultaneously, including high transparency in the visible light region, high efficient absorption in the whole UV range, photocatalytic activity, excellent resistance against washing and heat, and excellent mechanical properties such as high coating adhesion, abrasion resistance to stabilize and improve PSC device performance. Photocatalytic activity using ZnO and AZO, along with ultraviolet light radiation, can lead to the removal of organic pollutants and, thus, the recovery of the coating to its original characteristics. The devices without the protective layer lost their initial efficiency after four days (9 h per day with a high intensity of 50 mW cm^–2^), while the devices with the protective layer of AZO and ZnO retained their initial performance of 40% and 30%, respectively during 11 days under the same conditions.

## Results and discussion

**Figure 1:**
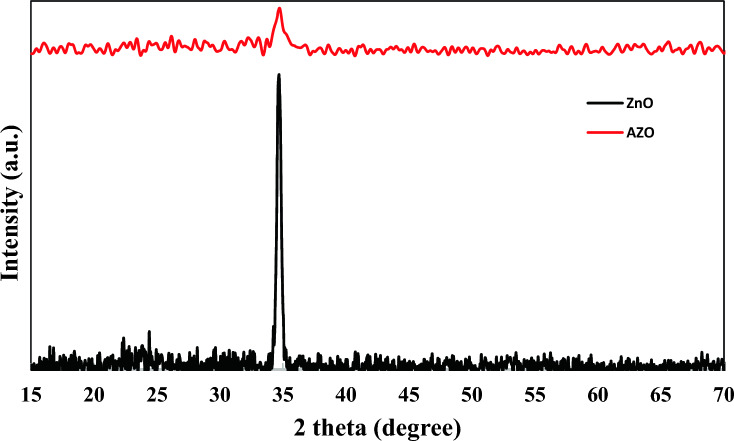
XRD Patterns of the pure ZnO and AZO thin films coated on the glass side of the FTO substrates.

Figure 1 shows the X-ray diffraction (XRD) pattern of the ZnO and AZO thin films on the glass substrate. The pure ZnO exhibits well-defined diffraction at 2θ = 34.5° corresponding to the wurtzite hexagonal phase of ZnO and the lattice plane of (002). The crystallinity of ZnO films is because of creating defects in the lattice site. The average crystallite size of the particles on the thin films of ZnO and AZO were 18.30 and 8.64 nm, respectively, as calculated by the Scherer equation^46^.

**Figure 2:**
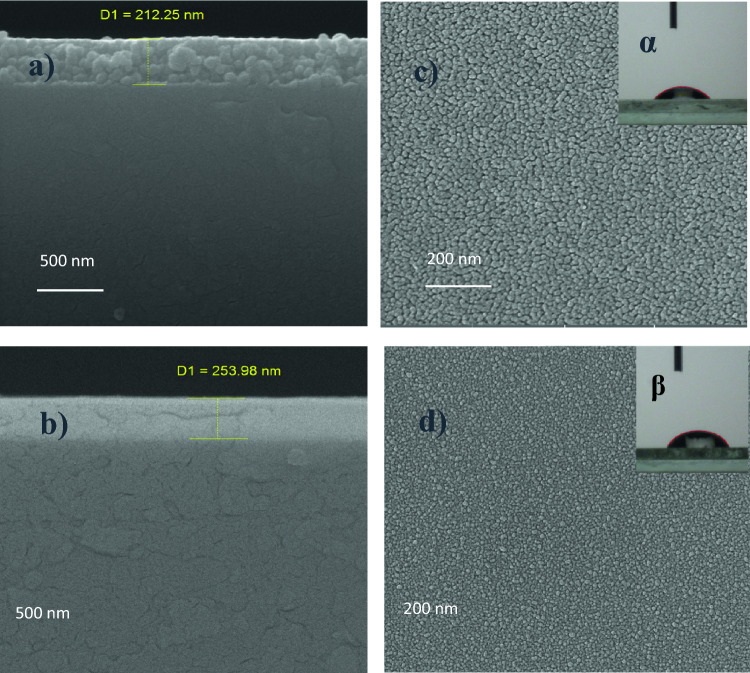
FE-SEM cross-sectional views of (**a**) ZnO and (**b**) AZO, and top-view images (**c**) ZnO and (**d**) AZO coated on the glass side of the PSC substrates. The inset images show a water droplet on the surface of ZnO (α), and AZO (β).

The top view of field emission scanning electron microscopy (FE-SEM) images indicate a uniform ZnO and AZO thin film architecture (Fig. 2c and d). The XRD spectrum shows that the particle size of AZO is smaller than that of ZnO. This suggests that substitution and shrinkage of Al^3+^ in the lattice structure of ZnO take place, reducing the grain size of AZO nanoparticles^47^. The insets display images of a water drop on the surfaces of the thin films of ZnO (Fig. 2c) and AZO (Fig. 2d) in the contact angle tests. The static contact angles of water for ZnO and AZO are 40.7° and 58.3°, respectively, indicating that the surfaces are in hydrophilic nature.

**Figure 3:**
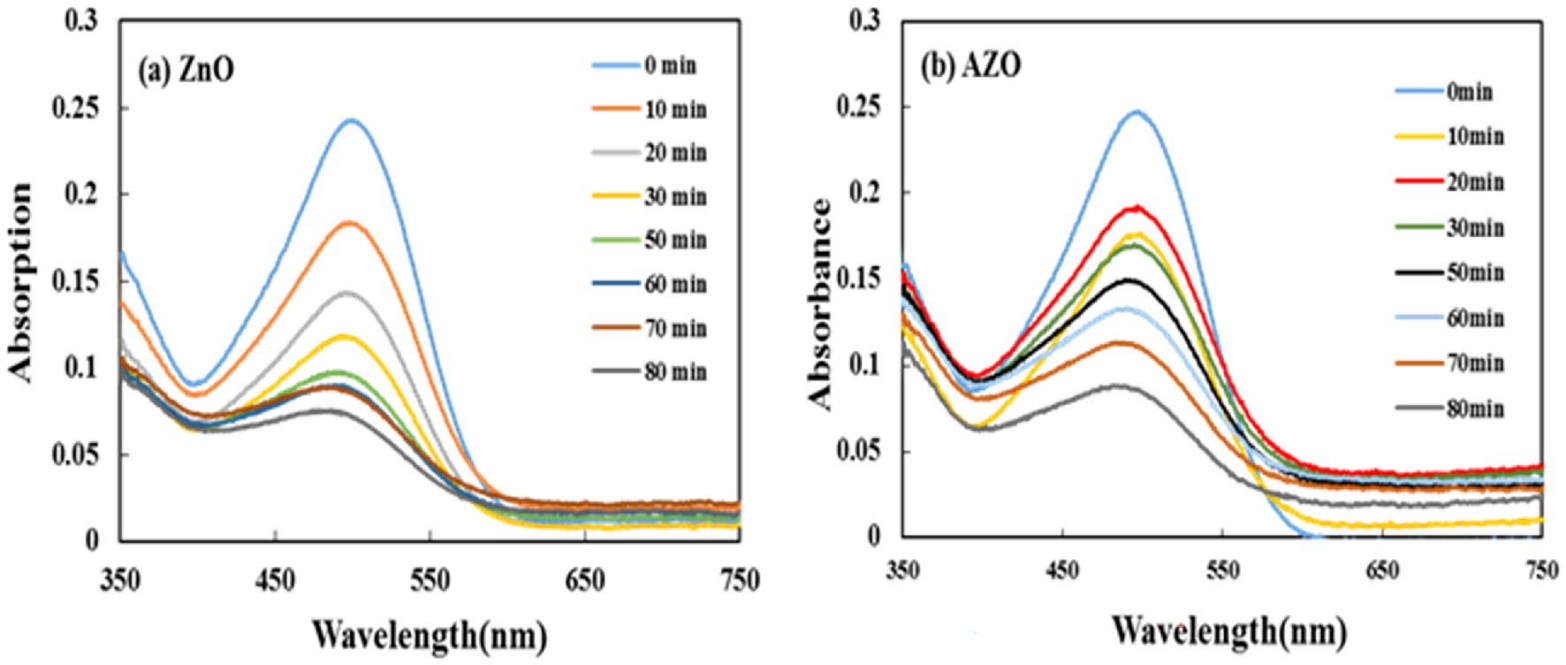
The decrease in the absorption intensity of congo red (CR) dye solution as a function of UV irradiation time in the presence of (**a**) ZnO and (**b**) AZO thin film as a catalyst.

Photocatalytic degradation of congo red (CR) under ultraviolet light irradiation was performed by deposition of 5 layers of AZO and ZnO on microscope slides (4 × 4 cm^2^) and placing of them in an aqueous congo red (CR) solution (5ppm). The absorption spectrum of the CR solution was measured at different time intervals. The changes related to the intensity of the absorption peak in λ_max_ = 506 nm indicate the degradation rate for ZnO is faster than AZO Fig. 3a and b. This could be related to the more hydrophilic nature of ZnO thin film leading to a significant decrease in pollutant molecules concentration after photoirradiation^47^.

**Figure 4:**
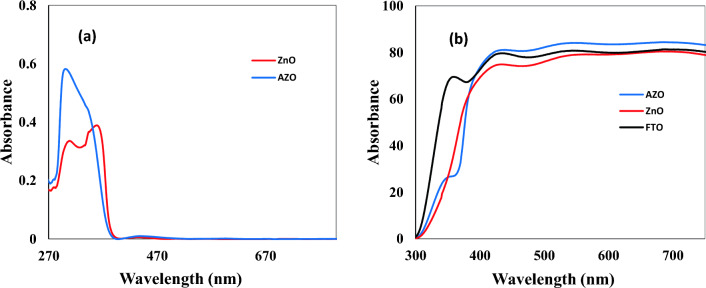
(**a**) UV–vis absorption and (**b**) transmission spectra of AZO and ZnO coatings compared with FTO blank (15 Ω sq^–1^).

The UV–visible absorption and transmittance spectra for fabricated multifunctional coatings on PSC substrate composed of ZnO (5 layers)/Glass/FTO and AZO (5 layers)/Glass/FTO have been shown in Fig. 4. As can be seen, these films thermally treated at 400 ºC present high absorbance in the 290–400 nm wavelength region and high transmittance in the visible wavelength region (400–700 nm). As can be seen from Fig. S1, Both AZO and ZnO films cover the UV spectral region of the sunlight well, although the absorption intensity of the former is higher. The transmission spectra of AZO and ZnO films clearly reveal the UV filtering feature and show that most visible light (more than 80%) can pass through the provided coatings. Furthermore, AZO coated on the glass side of the FTO substrate presents higher transmittance rather than FTO blank substrate and ZnO coated on the glass/FTO in the visible light range, which can be due to the lower refractive index (1.63) and higher porosity of AZO (34.3%) in comparison to ZnO (refractive index = 1.83 and porosity = 12.1%). Al doping of ZnO can reduce structural defects and modify the crystal structure of ZnO, which leads to a decrease in the refractive index of the material. As a result, the AZO layer has a lower refractive index than pure ZnO, which means that it can transmit more light without internal reflection or absorption through the material. In addition, AZO has a smaller particle size and a smoother surface than ZnO, which can increase light transmittance (Figs. 2, and S2)^40,41^. Thus, the perovskite material in the AZO/Glass/FTO/meso-TiO_2_/Perovskite configuration can absorb more visible light than the ZnO/Glass/FTO/meso-TiO_2_/Perovskite and Glass/FTO/meso-TiO_2_/Perovskite. Given that the UV light in the range of 290–380 nm is absorbed through UV blocker coatings of AZO and ZnO, perovskite photocatalytic degradation by TiO_2_ in the presence of UV filtering coatings of AZO and ZnO is suppressed compared to PSC without AZO and ZnO.

Since ultraviolet rays destroy the perovskite layer and reduce the stability of the solar cell, placing the ultraviolet light absorber layer on the outer surface of the solar cell or on the cover glass of perovskite modules prevents the ultraviolet light from entering the cell and the perovskite layer, and as a result improves solar cell stability. On the other hand, since these multi-purpose coatings are subject to physical and environmental damage, the more resistant these layers are to these damages, the less likely they are to be destroyed, thus increasing the stability of solar cells. Mechanical properties and resistance to environmental conditions of materials are mainly investigated by nanoindentation and nanoscratch tests. With the help of the nanoindentation test, the hardness of the coatings can be compared and with the help of the nanoscratch test, the wear resistance of the prepared coatings can be compared.

**Figure 5:**
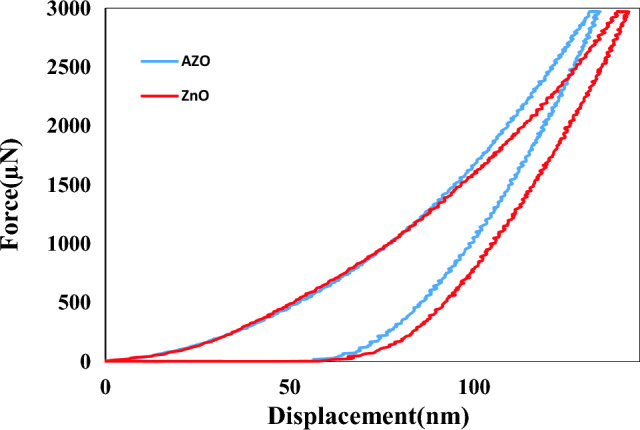
The nanoindentation curves wich determined Hardness and elastic module values for ZnO and AZO thin films.

**Figure 6:**
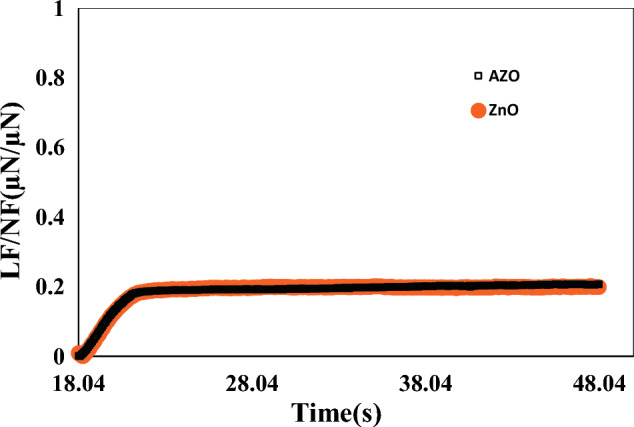
COF from nanoscratch experimental at a load of 2972 μN for the deposited coatings of ZnO and AZO.

The hardness and elastic modulus of the coating materials are shown in Table 1. The indentation curves of these films (maximum load 2972 μN) are represented in Fig. 5. The indentation depth at 2972 μN was ~ 138.6 nm for AZO and ~ 143 nm for ZnO coating, representing ~ 54.5% and ~ 67.3% of the total coating thickness, respectively. The Hardnesses of 5.07 GPa and 4.92 GPa were obtained for AZO and ZnO coatings, respectively. Elastic modulus was 79.8 for AZO and 73.7 GPa for ZnO. Larger grain size, from ~ 16.57 to ~ 19.1 nm, and porosity increase from 12 to 34% had a remarkable impact on the decrease of mechanical properties. Aluminium doping of ZnO can improve thin film mechanical properties in two ways: first, aluminium can be included in the crystal lattice structure of the Nizelak layer, which can lead to an increase in the density of the thin film, which can improve its mechanical properties. secondly, the addition of aluminium can affect the microstructure of the thin layer, for example, it can cause the formation of a more uniform and compact layer, which can improve the resistance against cracking and delamination^43,44^. Wear is defined as a set of adhesion, transfer, abrasion, fatigue, and oxidation. The levels of frictional resistance are impacted by the coefficient of friction (COF). In other words, A lower friction coefficient increases wear resistance^37,40^. Figure 6 shows the COF data of AZO and ZnO coatings at the scratch load of 2968 μN. As shown, the COFs for AZO and ZnO coating are low (~ 0.2) and nearly constant. The low friction coefficient of these coatings indicates the high resistance of these coatings against wear. Since cover glasses are often exposed to rough conditions, the AZO and ZnO deposited thin films can be used on cover glass due to having suitable mechanical and optical properties.

**Table 1:** The mechanical parameters of the thin films of AZO and ZnO (5 devices for case).

Coating material	Hardness	Er(GPa)	Contact stiffness	COF
AZO	11.51 ± 0.39	123.9 ± 2.47	71 ± 1.13	0.2
ZnO	10.61 ± 0.15	105.1 ± 2.11	62.8 ± 1.7	0.2

**Figure 7:**
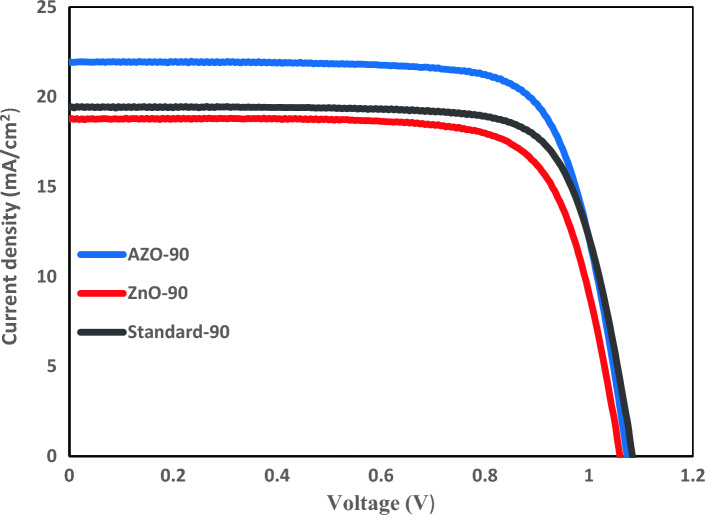
The *J–V* curves of the best PSCs with and without AZO and ZnO.

The photocurrent density–voltage (*J-V*) plots of the best PSC devices with and without UV absorber films are shown in Fig. 7 and Table 2. Applying the multifunctional films of AZO on the front of PSC increases the *Jsc* from 19.40 for PSC without UV filtering coating to 21.94 mA cm^-2^ for PSC-based AZO. The more transmittance of visible light through AZO/Glass/FTO compared with Glass/FTO leads to a higher absorption of light by perovskite materials and consequently enhanced current density. The AZO coating allows more visible light to pass into the PSC than the PSC without the UV-absorbing layer. The more visible light the perovskite absorber layer receives, the higher the PSC performance.

**Table 2:** The average PV parameters of the PSC-based AZO, PSC-based ZnO, and PSC without UV absorber layer under standard AM1.50 G illumination.

Device	JSC (mA cm^–2^)	Voc (v)	FF	PCE (%)
PSC based AZO	21.17 $\pm$ 0.86	1.0681 $\pm$ 0.0023	0.7503 $\pm$ 0.0078	16.97 $\pm$ 0.68
PSC based ZnO	19.96 $\pm$ 0.90	1.0582 $\pm$ 0.0047	0.7448 $\pm$ 0.0073	15.72 $\pm$ 0.71
PSC without UV absorber layer	19.11 $\pm$ 0.40	1.0783 $\pm$ 0.0023	0.7536 $\pm$ 0.0130	15.53 $\pm$ 0.36

The *Jsc* raising resulting from increased light transmittance has led to a PCE enhancement from 16.02 to 17.74% for AZO, but a downturn from 16.02 to 14.81% for ZnO was obtained due to decreased transparency of FTO substrate by a coating of ZnO.

We investigated the stability of both PSCs without and with UV blocker films for 11 days in a dry glove box at room temperature after the 9 h of UV light irradiation with the intensity of 50 mW cm^–2^ per day for 11 days. Since the power of UV light (at wavelengths below 400 nm) within the AM1.5 solar spectrum is 4.6 mW cm^–2^, thus in the present study, the UV illumination intensity (50 mW cm^–2^) is more than ten times the solar UV irradiation. The PSC devices without AZO and ZnO coatings in this UV-induced aging test (Fig. 7) failed after four days of exposure, but all cells with AZO and ZnO coatings maintained under the same conditions, 40% and 30% of their initial PCE after 11 days respectively. Furthermore, AZO has created higher stability than others due to a higher UV absorption.

Furthermore, to investigate how the presence of these coatings affects the performance of the device in different angles, photovoltaic parameters were measured at three different angles 90˚, 60°, and 30° (Figs. S3–S5 and Tables S1–S4). As can be seen, when the incident angle of simulated solar light decreases from 90˚ to 30˚, the short circuit current density (Jsc) decreases from 21.94 to 3.46 mA cm^2^ for PSC-based AZO, 18.74 to 3.33 mA cm^2^ for PSC-based ZnO and 19.40 to 2.76 mA cm^2^ for PSC without UV absorber layer (as stated in Table S1). The highest PCE is achieved when the device is positioned horizontally, perpendicular to the light source at a 90˚ angle. These findings suggest that achieving the highest possible power conversion efficiency is only feasible when the solar cell is situated in a horizontal position under the intensity of 1 sun illumination (AM1.5).

## Preparation of multifunctional coatings

### Preparation of ZnO and AZO thin films

First, a ZnO sol and the related film were prepared in the following way. A 0.6 M solution containing 2750 mg of zinc acetate di-hydrate (Merk 99%) in 25 ml 2-propanol(Merck, 99.9%) was prepared by using a magnetic stirrer for 60 min at 68 ºC. The solution became opaque immediately, then diethanolamine (DEA) (Merk,99%) was added dropwise to stabilize the sol. In this regard, The molar ratio of Zn^2+^ /DEA was kept 1:1. To prepare AZO sol, first the sol of ZnO was prepared as mentioned, then 400 mg of aluminium nitrate nonahydrate (Riedel-de Haën, 99%) was added to the sol. The molar ratio of the dopant (aluminium nitrate nonahydrate) in solution [Zn/Al] was 8%.

The solutions were stirred for 60 min at 68 ºC to form homogenous solutions**.** The solutions were deposited on the glass side of the pre-cleaned FTO substrate by dip coating process in withdrawal rates of 50 and 40 mm min^–1^ for ZnO and AZO, respectively. After each coating, to remove the solvent and the remaining organic residuals, the films were heated for 15 min at 300 ºC. This coating method was repeated five times to reach its desired layer thickness. Finally, the dried films were inserted into a furnace and annealed at 400 °C for 1 h under air with a heating rate of 1 °C min^–1^.

## Fabrication of perovskite solar cells

The following process fabricated the perovskite photovoltaic cells. First, FTO-coated glass substrates were etched with zinc powder and 30% hydrochloric acid (Merk,37%) to form a detached electrode pattern. After that, a TiO_2_ compact layer was deposited by spray pyrolysis method on the patterned FTO substrates from a precursor solution containing 9 mL of anhydrous ethanol (Merk, 99.9%), 0.6 mL of titanium di-isopropoxide (Merk, 99.9%) and 0.4 mL of bis acetylacetonate (Merk, 99.9%)) and then annealed at 450 °C for 30 min. A mesoporous TiO_2_ layer was deposited on the TiO_2_ compact layer by a spin coating method (50 µl for 14 × 14 mm) at 4000 rpm for 20 s by using a TiO_2_ paste (Sharif Solar with 30 nm average particle size) diluted in ethanol (1:5, weight ratio). Then, the substrates were immediately dried at 100 °C for 10 min and sintered at 500 °C for 30 min under ambient conditions. The perovskite precursor solution was prepared by dissolving MAI (Merck, 99.9%), PbI_2_ (Merck, 99.9%), and DMSO (Merck, 99.9%) (1:1:1 mol %) in DMF (Merck, 99%, 50 wt %). The perovskite solution was spin-coated by a one-step spin-coating method at 4000 rpm for 50 s (50 μL for 14 × 14 mm), and the substrates were treated with chlorobenzene at 10s after the start of spin coating. The spiro-OMeTAD-based hole transporting layer was coated via spin-coating method at 4000 rpm for 50 s where spiro-OMeTAD (Merk, 99%)/chlorobenzene (72 mg/ml) solution was employed with the additives containing 17.5 µl of a stock solution (520 mg Li-TFSI in 1 mL acetonitrile) in 1mL of chlorobenzene and 28.8 µL of 4-tert-butylpyridine^48,49^. Finally, 80 nm gold as the back electrode was thermally evaporated on the HTM layer under a high vacuum. The active area of the PSC was 0.09 cm^2^.

## Characterization

The J-V curves were measured under a solar simulator (Sharif Solar, AM 1.5G, 100 mW cm^–2^) illumination. The UV–vis absorption and transmittance spectra of prepared thin films were recorded by UV–vis spectroscopy (V-670) in the wavelength range of 190–900 nm. The surface morphology and thickness of the thin films were analyzed by Field emission scanning electron microscopy (FE-SEM, S4160 Hitachi Japan). The crystalline phase of the AZO and ZnO films was characterized using XRD analysis using a Bruker, D8ADVANCE. The porosity and the refractive index of the AZO and ZnO thin film were measured using an ellipsometer (Senteck se800). The water contact angles of the coated films were determined via Pendant Drop contact angle and IFT Measurement (Fars EOR Technologies). The hardness, elastic modulus, and contact stiffness of the deposited films were measured by a nanoindentation tester (Berkovich). These films' coefficient of friction (COF) was examined with Berkovich diamond indenter by scratch test utilizing a Berkovich Nanoscratch Tester with μm radius spheroconical diamond indenter.

**Figure 8:**
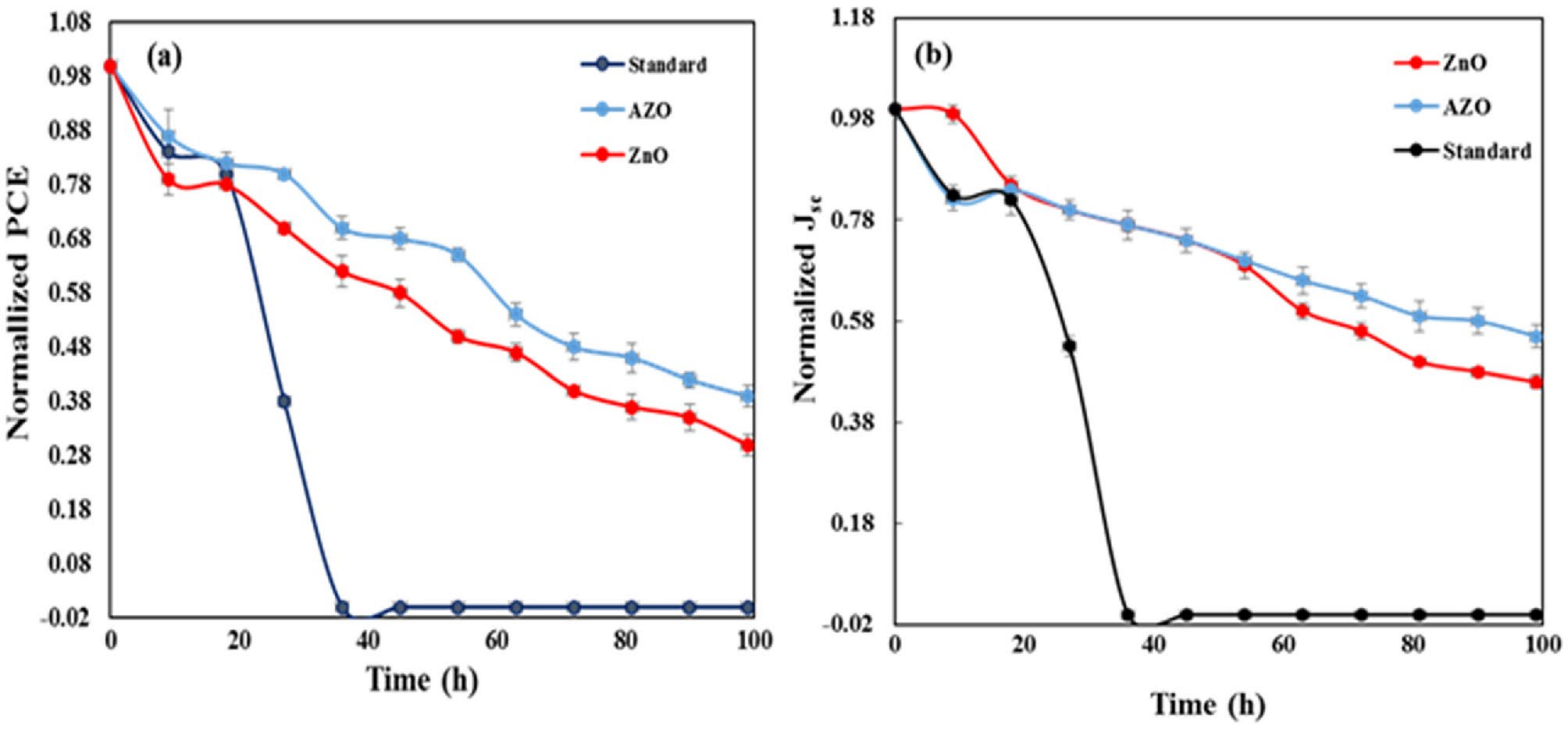
Normalized stability evaluations of the different PSCs, including AZO, ZnO, and FTO, without UV absorber. (**a**) PCE and (**b**) J_sc,_ (3 devices for each case).

To improve the stability of the PSCs against ultraviolet radiation and mechanical conditions, two types of multifunctional coatings composed of AZO and ZnO were deposited on the front side of the PSC devices. The thickness of the layers was optimized to maximize UV light absorption and visible light passing. The multifunctional coatings exhibited several properties simultaneously, including high transparency in the visible light region, highly efficient absorption in the whole UV range, excellent resistance against washing and heat, and excellent mechanical properties of the coatings such as high hardness and low coefficient, which leads to increased resistance against wear. The photocatalytic self-cleaning properties of these prepared coatings make them stable against environmental pollutants. The effect of the AZO and ZnO on UV stability was examined in an ambient atmosphere (Fig. 8). With AZO and ZnO, the devices maintained 40% and 30% of their initial efficiency for 11 days (9 h per day) against the UV light with a high intensity of 50 mW cm^–2^. Moreover, due to having suitable mechanical and optical properties, the AZO and ZnO deposited thin films can be used as cover glasses for all kinds of solar cells. In this regard, AZO can be a more appropriate candidate.

### Supplementary Information

Supplementary Information.

## Data Availability

All data generated or analysed during this study are included in this published article and its supplementary information files.
