# Supplementary material for: Stability enhancement of perovskite solar cells using multifunctional inorganic materials with UV protective, self cleaning, and high wear resistance properties
Source: Sci Rep. 2024 Mar 18;14:6466. doi: 10.1038/s41598-024-57133-8 (PMC10948775; doi:10.1038/s41598-024-57133-8)
Supplement: Supplementary file 1 — Supplementary Information. [file 41598_2024_57133_MOESM1_ESM.docx]

**stability enhancement of perovskite solar cells using multifunctional inorganic materials with UV protective, self cleaning, and high wear resistance properties**

Seyyedeh Sedigheh Azad, Reza Keshavarzi,* Valliolah Mirkhani,* Majid Moghadam, Shaham Tangestaninejad, Iraj Mohammadpoor-Baltor

*Department of Chemistry, University of Isfahan, Isfahan 81746-73441, Iran*


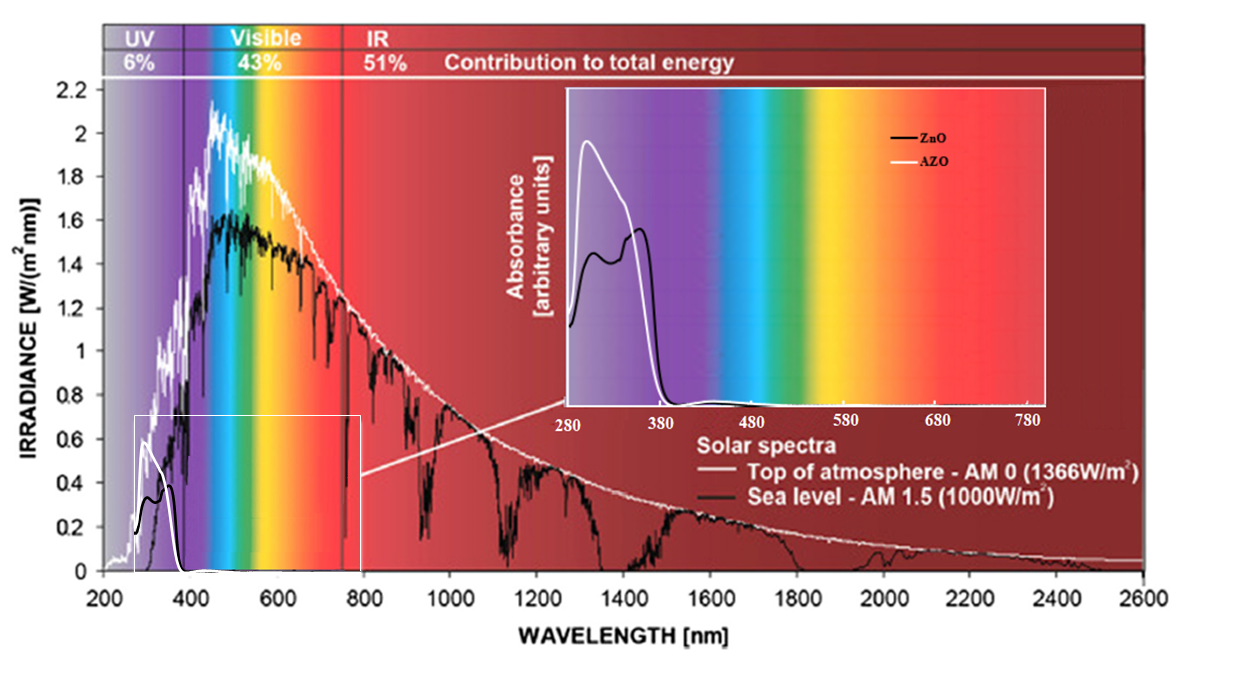


Figure S1: Absorption spectra of AZO and ZnO thin films in solar irradiance spectrum


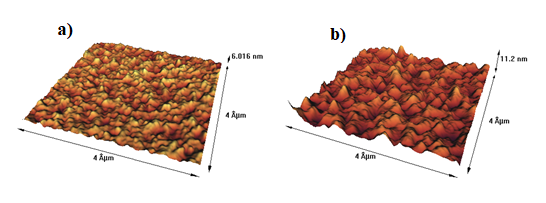


Figure S2: Atomic force microscopy (AFM) images of AZO (a) and ZnO (b) thin films. Roughness values for AZO and ZnO thin films were 0.73 nm and 1.01 nm, respectively.

Figure S3: The *J–V* curves of the PSC-based AZO in different angles of irradiation relative to the light source

Figure S4: The *J–V* curves of the PSC-based ZnO in different angles of irradiation relative to the light source

Figure S5: The *J–V* curves of the PSC without UV absorber layer(standard) in different angles of irradiation relative to the light source

Table S1. Short circuit current density **(**JSCs **(**mA cm^-2^**))** of the PSC-based AZO, PSC-based ZnO, and PSC without UV absorber layer in different angles of irradiation relative to the light source.

| Device | 90 | 60 |  | 30 |
| --- | --- | --- | --- | --- |
| PSC based AZO | 21.94 | 6.21 |  | 3.46 |
| PSC based ZnO | 18.74 | 5.95 |  | 3.33 |
| PSC without UV absorber layer | 19.40 | 5.86 |  | 2.76 |

Table S2. Open circuit voltage **(**Vocs (V)) of the PSC-based AZO, PSC-based ZnO, and PSC without UV absorber layer in different angles of irradiation relative to the the light source.

| Device | 90 | 60 |  | 30 |
| --- | --- | --- | --- | --- |
| PSC based AZO | 1.06 | 1.01 |  | 0.99 |
| PSC based ZnO | 1.05 | 1.01 |  | 0.99 |
| PSC without UV absorber layer | 1.07 | 1.02 |  | 0.99 |

Table S3. Fill factors of **(**FFs) of the PSC-based AZO, PSC-based ZnO, and PSC without UV absorber layer in different angles of irradiation relative to the light source.

| Device | 90 | 60 |  | 30 |
| --- | --- | --- | --- | --- |
| PSC based AZO | 0.75 | 0.78 |  | 0.77 |
| PSC based ZnO | 0.75 | 0.73 |  | 0.70 |
| PSC without UV absorber layer | 0.76 | 0.78 |  | 0.80 |

Table S4. Power conversion efficiency (PCEs (%)) of the PSC-based AZO, PSC-based ZnO, and PSC without UV absorber layer in different angles of irradiation relative to the light source.

| Device | 90 | 60 |  | 30 |
| --- | --- | --- | --- | --- |
| PSC based AZO | 17.74 | 4.97 |  | 2.69 |
| PSC based ZnO | 14.81 | 4.62 |  | 2.56 |
| PSC without UV absorber layer | 16.02 | 4.72 |  | 2.22 |
